# Identification of direction in gene networks from expression and methylation

**DOI:** 10.1186/1752-0509-7-118

**Published:** 2013-11-01

**Authors:** David M Simcha, Laurent Younes, Martin J Aryee, Donald Geman

**Affiliations:** 1 Department of Biomedical Engineering, Johns Hopkins University, Baltimore, MD 21218, USA; 2 Department of Applied Mathematics and Statistics, Johns Hopkins University, Baltimore, MD 21218, USA; 3 Department of Pathology, Harvard Medical School, Boston, MA 02115, USA; 4 Department of Pathology, Massachusetts General Hospital, Charlestown, MA 02129, USA; 5 Department of Applied Mathematics and Statistics and Institute for Computational Medicine, Johns Hopkins University, Baltimore, MD 21218, USA

**Keywords:** Gene regulation, Methylation, Microarrays, Bayesian networks

## Abstract

**Background:**

Reverse-engineering gene regulatory networks from expression data is difficult, especially without temporal measurements or interventional experiments. In particular, the causal direction of an edge is generally not statistically identifiable, i.e., cannot be inferred as a statistical parameter, even from an unlimited amount of non-time series observational mRNA expression data. Some additional evidence is required and high-throughput methylation data can viewed as a natural multifactorial gene perturbation experiment.

**Results:**

We introduce IDEM (Identifying Direction from Expression and Methylation), a method for identifying the causal direction of edges by combining DNA methylation and mRNA transcription data. We describe the circumstances under which edge directions become identifiable and experiments with both real and synthetic data demonstrate that the accuracy of IDEM for inferring both edge placement and edge direction in gene regulatory networks is significantly improved relative to other methods.

**Conclusion:**

Reverse-engineering *directed* gene regulatory networks from static observational data becomes feasible by exploiting the context provided by high-throughput DNA methylation data.

An implementation of the algorithm described is available at http://code.google.com/p/idem/.

## Background

As the analysis of high-throughput gene expression data, notably phenotypic classification of samples [1-7], has expanded and matured, the focus has begun to shift towards mechanism and systems modeling [8-10]. In particular, much of the unrealized value of high-throughput molecular data may be in increasing our understanding of how various molecules interact in vivo, i.e., by reverse-engineering biological networks, hopefully revealing how disease states form and what targets might be available for their treatment. In the case of transcript data the most relevant type of network is one modeling transcriptional regulation. This may be thought of as a causal graph, wherein each node represents a variable and a directed edge is placed from every cause to each of its direct effects. From a causal gene regulatory graph, one then infers the effects of under- or over-expressing the mRNA level of one gene on the mRNA expression of other genes. The definition in terms of mRNA expression is a pragmatic choice, as this can be easily measured in a high-throughput fashion and can serve as a surrogate for protein concentration under some circumstances [11].

**Figure 1:**
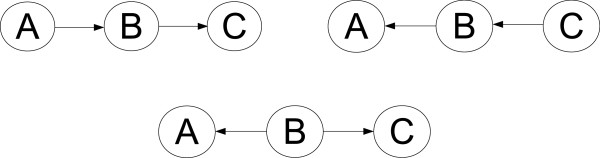
**Causal Vs. Bayesian networks.** Three DAGs with different meanings when interpreted as causal graphs but identical meanings when interpreted as Bayesian networks. As Bayesian networks, all three graphs represent different forms of the same probability factorization, namely *A* ⊥ *C*|*B*. Therefore, the correct causal graph cannot be identified statistically by examining independence relationships among variables.

Whereas causality is not a statistical concept, there is an important relationship between causal graphs and Bayesian networks, which are stochastic graphical models commonly used to represent large-scale biological networks, in particular gene regulatory networks. Bayesian networks are probability distributions over directed acyclic graphs (DAG) such that each node represents a variable and each variable is statistically independent of its non-descendents given its parents. The connecting concept between causal graphs and Bayesian networks is the Causal Markov Condition [12,13], which states that a variable is independent of its non-effects given all of its direct causes. If a complete causal DAG (one that includes all common causes of any pair of variables) is interpreted as a Bayesian network, the statistical properties of the system will be correctly represented. Despite this relationship, the mapping from a Bayesian network graph to a causal DAG is non-trivial; see Methods. For one thing, multiple Bayesian network DAGs can map to the same independence relationships. In other words, these models are not statistically “identifiable” and multiple causal situations represented by different DAGs can map to a single set of independence assumptions. One example of this is shown in Figure 1.

Because of this non-identifiability, the central challenge in reverse-engineering a gene regulatory network from observational data is placing *directed* edges: determining the direction of the causal arrow between a pair of genes that are irreducibly statistically dependent and believed at least provisionally to be causally related. However, an isolated causal assumption cannot be tested using only observational data [14]. Therefore, most past attempts to reverse-engineer gene regulatory networks using expression data fall into one of three categories. The first category of methods requires time series data and assumes that cause will temporally preceed effect, examples being dynamic Bayesian networks [15,16], Granger causality [17] or a similar time shifted correlation technique [18]. The second category uses techniques such as ordinary differential equations and requires targeted perturbation of specific genes in quantitatively well-defined ways [19]. Compounding these difficulties, time series or perturbation data is often difficult to obtain, either for ethical or technical reasons, from human *in vivo* biological states. Methods in the third category utilize static data but allow edge directions to remain unidentifiable for many or all subgraphs; these include information-theoretic algorithms [20,21], decision tree based algorithms [22], and static Bayesian network algorithms [23-25].

Our approach to dealing with causal direction is to broaden the context beyond mRNA expression and extract information from high-throughput data about auxiliary variables associated with each gene. We use causal assumptions which are justified on biological rather than computational grounds about connections in the extended network between the genes and the auxiliary variables. Finally, we then test whether the observed data is consistent with additional causal assumptions under the Causal Markov Condition. Even though a causal assumption cannot be tested in isolation using observational data, a set of causal assumptions can yield predictions that can be tested using such data [14]. We use methylation data in this study to illustrate our approach although other choices are possible. In mammalian cells, DNA methylation in the promoter region of a gene is frequently used as an epigenetic gene silencing signal. Notably, changes in promoter region methylation appear to cause targeted, gene-specific effects. For example, methylation appears to play a role in maintaining gene silencing in genomic imprinting [26]. Similarly, tumor suppressor genes are frequently hypermethylated in cancer [27,28]. Techniques have been recently developed for measuring methylation in a high-throughput fashion [29]. In many cases, such measurements provide the context necessary to make edge directions identifiable when reverse-engineering gene regulatory networks.

This context is exploited by building enhanced directed regulatory network using two types of nodes for each gene: conventional ones representing mRNA expression and others representing methylation levels, both measurements being obtained from non-time series, high-throughput, observational data. A key simplifying assumption usually made in methodologies designed for large-scale reverse-engineering [20,21], including ours, is that biological interactions among genes (such as regulator-target relationships) imply statistical dependence at a pairwise level. Therefore, for every pair of genes with a significant statistical interaction, we construct a simple, four-variable Bayesian network representing two mRNA variables (i.e., two genes) and two corresponding methylation states. This construction includes estimating the direction of the arrow between the two genes using a likelihood ratio test. In effect, the resulting algorithm, IDEM (Identification of Direction from Expression and Methylation) can be thought of as a taking advantage of a natural multifactorial gene perturbation experiment. When a gene promoter region becomes differentially methylated across samples, the expression of the target gene may be perturbed. Measuring promoter region methylation can be thought of as measuring how the system has been perturbed. A key model assumption, therefore, is that methylation of the promoter region of a gene directly affects the mRNA expression of the downstream gene and does not directly causally affect the expression of any other gene. Under this assumption, discovering the direction of a causal edge from non-time series observational data becomes possible in some cases, especially in subnetworks that are acyclic (tree-like). (Details are in the Theoretical results section).

Our main contribution is then a simple and novel statistical test to determine the direction of regulatory edges from the joint distribution of methylation and mRNA expression data. No time-series or intervention data is used. We explore the conditions under which this test is consistent from a theoretical perspective and measure its accuracy empirically using both real and simulated data, demonstrating that causal gene regulatory networks can at least be partially inferred from static observational data provided there is sufficient auxiliary information about the regulatory context.

## Methods

### Data acquisition and pre-processing

Expression and methylation data were obtained from The Cancer Genome Atlas [30] (TCGA). The expression platform chosen was the Agilent G4502A_07, and the methylation platform was the Illumina Infinium HumanMethylation27 panel. Approximately 12,000 genes (depending on how many probes were discarded due to missing data) are common to both platforms. The sample sizes and number of available genes for these datasets are shown in Table 1. All ovarian serous cystadenocarcinoma (Ovarian) and glioblastoma multiforme (GBM) patient samples containing data for both platforms were used. We omit detailed results for the colon adenocarcinoma, breast invasive carcinoma and lung squamous cell carcinoma samples even though methylation and expression data are available for these datasets because the much smaller sample sizes result in very few significant edges being found and poor accuracy among the edges that are found.

**Table 1:** Sample size

**Dataset**	**N samples**	**N genes**
GBM	279	11834
Ovarian	536	11270

The sample sizes of the TCGA datasets used.

Where technical replicates existed, the values were averaged. Probes for which data was missing for any sample were discarded. To reduce the severity of batch effects [31], the batch-specific mean expression or methylation value for each gene was subtracted out, and then the global mean added back. Thus, all batches were forced to have the same mean for any given gene. Both expression and methylation data were then transformed to rank space on a per-sample basis. Finally, to simplify computations involving mutual information, each probe was binned into *B* equal frequency bins. Where multiple probe sets (for expression or methylation) mapped to the same gene, the pair of probe sets (one for methylation, one for expression) with the highest mutual information was selected to represent that gene. Preprocessed data can be found in Additional files 1, 2, 3 and 4.

### Causal graphs and Bayesian networks

As indicated earlier, the relationship between causal graphs and Bayesian networks is complex. First, the Causal Markov Condition holds for a causal graph *G* containing vertices *V* only if all common causes of any pair of variables in *V* are included in the set of vertices *V*[12]. Marginalizing over common causes excluded from *V* can introduce statistical dependencies not predicted by the Causal Markov Condition as applied to *G*. Even under the strong assumptions that all common causes are measured (causal sufficiency [12]) and that no independences not implied by the causal DAG and Causal Markov Condition are present (causal faithfulness [12,13]), the Causal Markov Condition does not imply that a Bayesian network graph that accurately describes the independence relationships among the variables under study accurately represents causality when interpreted as a causal graph. Finally, there is the identifiability problem illustrated in Figure 1.

**Figure 2:**
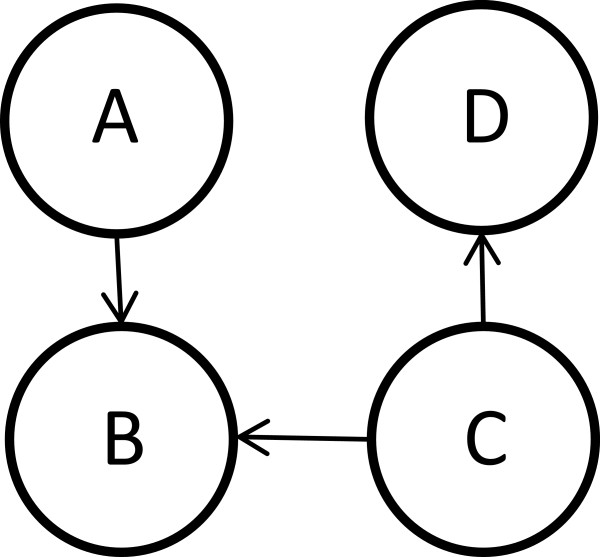
**Identifiability.** A hypothetical four-gene network for which a subset of causal edge directions might be statistically identifiable under the Causal Markov Condition only from non-time series observational expression data assuming all edge placements are correct. If treated as a Bayesian network, this graph represents the probability distribution factorization *P*(*A*, *B*, *C*, *D*) = *P*(*A*)*P*(*C*)*P*(*D*|*C*)*P*(*B*|*A*, *C*). The directions of the edges in the subnetwork {*A*, *B*, *C*} may be identifiable, as no other subgraph with these edge placements could produce a situation such that *A* is absolutely independent of *C* but these variables may become dependent when conditioned on *B*. However, the direction of the *C* - *D* edge is not identifiable. Reversing it would produce a different form of the same factorization as the direction shown.

Note, however, that causal direction may be identifiable under the Causal Markov Condition for some subgraphs from static data alone given correct edge placement; see Figure 2 for illustration of such a scenario. For a more thorough discussion of this issue we refer the reader to [23].

### Statistical framework

As mentioned above, a standard assumption in learning regulatory networks is that the mRNA levels of pairs of genes which are biologically interacting are dependent statistically as random variables. Plausible scenarios where this assumption is untrue do exist. For example, if a gene is regulated by the interaction of two regulators via XOR logic (activation if both regulators are active or if both are inactive and inhibition otherwise), then the gene can be independent of each of its regulators taken individually. On the other hand, the number of parameters to be estimated increases exponentially in the order (binary, ternary, etc.) of interactions to be learned. Therefore, we argue that for realistic sample sizes the bias error created by ignoring these higher-order scenarios will likely be smaller than the variance error suffered by attempting to recover them. It’s also important to note that statistical dependence alone does not imply causal dependence, for example if the expression of two genes has a common regulator or hidden variable. We attempt to mitigate this with the non-causal (NC) pruning and data processing inequality steps detailed later in this section.

Let *G* be the set of all genes for which both mRNA expression and promoter region methylation data are available. For any gene *g* ∈ *G*, let *M*_*g*_ be the promoter region methylation of gene *g* and let *E*_*g*_ be the mRNA expression level of gene *g*. As is common practice, the mutual information *I*(*X*;*Y*) between two random variables *X*, *Y*[32] will serve as a test statistic for independence, recalling that *X* ⊥ *Y* if and only if *I*(*X*;*Y*) = 0. Similarly, for three random variables *X*, *Y*, *Z*, we have *X* ⊥ *Y*|*Z* if and only if *I*(*X*;*Y*|*Z*) = 0. (Here and in the rest of this paper, we will use the standard notation *X* ⊥ *Y* to indicate that variables *X* and *Y* are independent and *X* ⊥ *Y*|*Z* to indicate that they are conditionally independent given a third variable, *Z*).

The first step of IDEM is to construct a mutual information relevance network [21] for mRNA expression. This means placing an undirected edge between every pair of genes *G*_1_ and *G*_2_ if the empirical evaluation of the mutual information of their mRNA expression (that we will denote $Î(E_{1};E_{2})$) exceeds a threshold and concluding that *E*_1_ and *E*_2_ are not statistically independent. Using the fact that, under null hypothesis that *E*_1_ ⊥ *E*_2_, Wilks’ Theorem [33] implies that $2\mathrm{NÎ}(E_{1},E_{2})$ approximately follows a chi-square with (*B* - 1)^2^ degrees of freedom where *N* represents the sample size, we place an undirected edge between *E*_1_ and *E*_2_ when the corresponding p-value is less than some value *α*.

### Local Bayesian network

Let *g*_1_, *g*_2_ be the two genes for which the hypothesis of statistical independence has been rejected. These are linked in the relevance network by a nondirected edge. Let *E*_1_, *E*_2_ be their mRNA expression levels and *M*_1_, *M*_2_ be their methylation levels in the measured parts of their promoter regions. Since methylation of several genes might be influenced by a single hidden variable, such as a methyltransferase mutation or environment, we also postulate a hidden (possibly multidimensional) variable *V* that may affect both *M*_1_ and *M*_2_. *V* is a theoretical construct; its exact nature is both unknown and unimportant. We now specify two competing local Bayesian network models for the joint distribution of (*E*_1_, *E*_2_, *M*_1_, *M*_2_, *V*). First, denote the (true) underlying joint distribution by

$$\begin{array}{ll} \;P(e_{1},e_{2},m_{1},m_{2},v) & =P(E_{1}=e_{1},E_{2}=e_{2},\\ & \;M_{1}=m_{1},M_{2}=m_{2},V=v) \end{array}$$

where we can assume all variables except *V* take values in {1, …, *B*}. For simplicity we will write *p*(*e*_1_) for *P*(*E*_1_ = *e*_1_), *p*(*e*_1_|*e*_2_) for *P*(*E*_1_ = *e*_1_|*E*_2_ = *e*_2_), and so forth. The meaning of all marginal and conditional distributions should be clear from the context.

**Figure 3:**
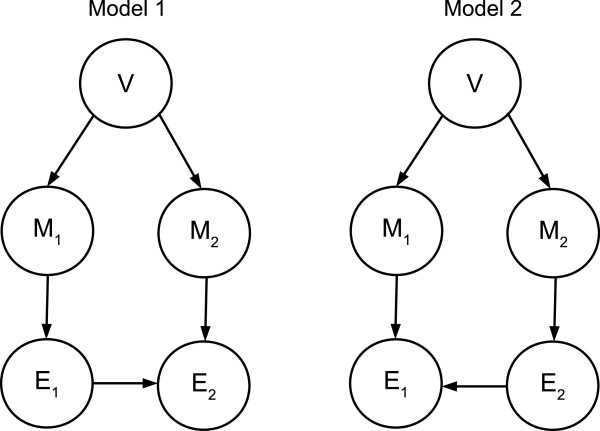
**Competing models.** The two direct causal models explaining the existence of significant mutual information between *E*_1_ and *E*_2_. These local models are interpreted as causal networks and as Bayesian networks via the Causal Markov Condition. *V* represents any hidden variables, such as methyltransferases, that may affect both *M*_1_ and *M*_2_. For each pair of genes with a putative edge, one of these models is selected using a likelihood ratio test.

Under the provisional assumption of a causal edge between *E*_1_ and *E*_2_, our objective is to determine which direction best explains the data. Ideally, this direction would be determined for a sufficiently large amount of data (sufficiently many realizations of of *E*_1_, *E*_2_, *M*_1_, *M*_2_) under the Causal Markov Condition; in other words, the direction of the edge would be “identifiable” as a statistical parameter. For this purpose, we assume the following as possible, competing models, illustrated in Figure 3:

$$\;\begin{array}{ll} \;\text{Model 1}:Q_{1}(e_{1},e_{2},m_{1},m_{2},v) & =P(v)P(m_{1}|v)P(m_{2}|v)\\ & \;\times P(e_{1}|m_{1})P(e_{2}|e_{1},m_{2}) \end{array}$$

$$\;\begin{array}{ll} \;\text{Model 2}:Q_{2}(e_{1},e_{2},m_{1},e_{2},v) & =P(v)P(m_{1}|v)P(m_{2}|v)\\ & \;\times P(e_{2}|m_{2})P(e_{1}|e_{2},m_{1}) \end{array}$$

Of course, we can marginalize the *V* variable and write, for example,

$$Q_{1}(e_{1},e_{2},m_{1},m_{2})=P(m_{1},m_{2})P(e_{1}|m_{1})P(e_{2}|e_{1},m_{2}).$$

The key difference is between the two models how the methylation of one gene affects the expression of the other. In Model 1 we are assuming that *E*_1_ ⊥ *M*_2_|*M*_1_ and *E*_2_ ⊥ *M*_1_|*E*_1_, *M*_2_, whereas in Model 2 it is the reverse: *E*_2_ ⊥ *M*_1_|*M*_2_ and *E*_1_ ⊥ *M*_2_|*E*_2_, *M*_1_.

From biological knowledge about methylation we know that one of these models is the correct causal graph for {*M*_1_, *M*_2_, *E*_1_, *E*_2_} if a causal link exists between *E*_1_, *E*_2_. This model is also accurate as a Bayesian network graph if marginalizing over all other relevant variables in the full network (such as the expression and methylation of other genes) does not introduce any additional statistical dependencies among {*M*_1_, *M*_2_, *E*_1_, *E*_2_}. This is because the Causal Markov Condition assumes that all common causes are included in a model. However, only low-order analysis is statistically and computationally feasible for large gene regulatory networks.

### A likelihood ratio test for direction

Assuming that one of these models represents the statistical ground truth, conditions under which the correct model is statistically identifiable can be derived. Consider the expected log likelihood ratio *E*_*P*_(*llr*) where the log likelihood ratio is:

() $$\begin{array}{llll} \text{llr}\;(E_{1},E_{2},M_{1},M_{2}) & \;=\; & \log\;\left(\;\frac{Q_{1}(E_{1},E_{2},M_{1},M_{2})}{Q_{2}(E_{1},E_{2},M_{1},M_{2})}\;\right) & \\ & \;=\; & \log\;\left(\;\frac{Q_{1}(E_{1},E_{2}|M_{1},M_{2})P(M_{1},M_{2})}{Q_{2}(E_{1},E_{2}|M_{1},M_{2})P(M_{1},M_{2})}\;\right) & \\ & \;=\; & \log\;\left(\;\frac{P(E_{1}|M_{1})P(E_{2}|E_{1},M_{2})}{P(E_{2}|M_{2})P(E_{1}|E_{2},M_{1})}\;\right) & \end{array}$$

If *P* = *Q*_1_, then this expected value is the Kullback-Leibler divergence between *Q*_1_ and *Q*_2_ and is necessarily non-negative. Similarly, by symmetry, if *P* = *Q*_2_, this value is non-positive. In fact, it can be shown (seeTheoretical results) that

$$\begin{array}{ll} \;E_{P}(\text{llr}(E_{1},E_{2},M_{1},M_{2})) & \;=\;I(E_{2};M_{1}|M_{2})\;+\;I(E_{1};M_{2}|M_{1},E_{2})\\ & \;-(I(E_{1};M_{2}|M_{1})\\ & \;+I(E_{2};M_{1}|M_{2},E_{1})). \end{array}$$

This expression is the difference between two non-negative terms, and the first one vanishes if and only if *P* = *Q*_2_ and the second one vanishes if and only if *P* = *Q*_1_. Assuming that either *Q*_1_ or *Q*_2_ holds, this expression is zero if and only if we are in the intersection of the two model classes:

$$\;\begin{array}{ll} \;I(E_{2};M_{1}|M_{2}) & =0,\;\;I(E_{1};M_{2}|M_{1})=0,\;\;I(E_{2};M_{1}|M_{2},E_{1})\\ & =0,\;\;I(E_{1};M_{2}|M_{1},E_{2})=0. \end{array}$$

*In summary, assuming a local Bayesian network for which the Causal Markov Condition holds, and assuming E*_1_* and E*_2_* are causally linked, the direction of the edge between them is identifiable except in the degenerate case in which all four independence statements are true.*

We can now use the Law of Large Numbers to put this result into practice. Suppose our data consist of *N* sample observations {*e*_1, *i*_, *e*_2, *i*_, *m*_1, *i*_, *m*_2, *i*_}, *i* = 1, …, *N*. The classical likelihood ratio of the data under the two models is then

$$\rho =\overset{N}{\underset{i=1}{\prod }}\frac{P(e_{1,i}|m_{1,i})P(e_{2,i}|e_{1,i},m_{2,i})}{P(e_{2,i}|m_{2,i})P(e_{1,i}|e_{2,i},m_{1,i})}.$$

By the Law of Large Numbers, log*ρ* converges to *E*_*P*_*llr*(*E*_1_, *E*_2_, *M*_1_, *M*_2_) when the sample size goes to infinity. This is the test statistic for our test for edge direction. Except in the degenerate case mentioned above, the logarithm of *ρ* divided by *N* converges to a strictly positive value under Model 1 and a strictly negative value under Model 2. Hence: **IDEM Decision Rule:***Select Model 1 if ρ > 1 and Model 2 if ρ < 1.*

Another interpretation of this rule can be obtained by writing

$$\;\begin{array}{ll} \;E_{P}(\text{llr}) & =(H(E_{2}|M_{2})-H(E_{2}|E_{1},M_{2}))-(H(E_{1}|M_{1})\\ & \;-H(E_{1}|E_{2},M_{1}))\;=\;I(E_{1},E_{2}|M_{2})\;-\;I(E_{1},E_{2}|M_{1}). \end{array}$$

Consequently, IDEM places an oriented edge from *E*_1_ to *E*_2_ if and only if $Î(E_{1},E_{2}|M_{2})>Î(E_{1},E_{2}|M_{1})$, i.e., if *M*_1_ has a stronger effect in decoupling *E*_1_ and *E*_2_ than *M*_2_.

### Pruning

We now introduce two pruning criteria that will reduce the large number of edges typically returned by relevance networks. The first criterion attempts to detect dependencies between *E*_1_ and *E*_2_ that could be due to co-regulation induced by a third, unobserved, variable, while the second ones prunes triangles by removing their weakest edge based on the data processing inequality.

#### Non-causal pruning

**Figure 4:**
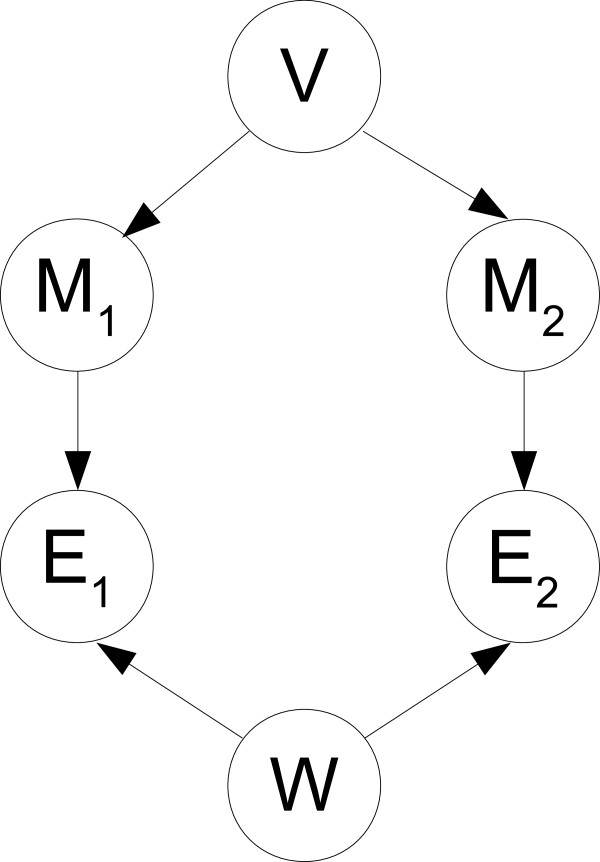
**Common causes.** A scenario where *E*_1_, *E*_2_ are regulated by a common cause *W* but do not causally affect one another.

After direction is determined, the edge may be eliminated via a non-causal (NC) pruning step if the mutual information between the methylation of the outgoing gene and the expression of the incoming gene is not significantly greater than zero given the methylation of the incoming gene. Our goal is to detect and discard situations in which the relationship between *E*_1_ and *E*_2_ is obtained via the causal effect of a third (possibly hidden) variable, say *W*, as depicted in Figure 4. For such causal relationship, one has *M*_1_ ⊥ *E*_2_|*M*_2_ and *M*_2_ ⊥ *E*_1_|*M*_1_. One of these is assumed to be true even if there is a direct causal relationship between *E*_1_, *E*_2_ depending on whether Model 1 or Model 2 is chosen in the likelihood ratio test. NC pruning tests whether the other of these can be statistically ruled out, e.g. if Model 1 is chosen in the likelihood ratio test then *I*(*M*_1_;*E*_2_|*M*_2_) significantly > 0. This is tested using Wilks’ theorem, where under the null hypothesis of conditional independence *M*_1_ is constrained to be independent of *E*_2_ at every level of *M*_2_. The *α* value used for this test is the same as the one used to build the relevance network. If the null hypothesis cannot be ruled out, the edge is removed. Beside removing common regulator cases, NC pruning also tends to remove edges for which the log likelihood ratio (| log*ρ*|) is close to zero and thus the confidence in the direction assigned is low.

#### Indirect edge removal

After edges are placed, maximum likelihood directions determined, and NC pruning is carried out, the graph is pruned using the data processing inequality method as introduced in ARACNE [20]. The goal here is to remove spurious edges, for which nonzero mutual information can be attributed to indirect interactions among modeled variables. For example, if Gene A regulates gene B and gene B regulates gene C, then *I*(*A*;*C*) may be strictly positive even if no direct regulatory link exists. Since feedback loops cannot be detected from non-time series observational data and inconsistencies in the likelihood ratio test in loopy scenarios prevent reliable detection of feed-forward loops, the most parsimonious explanation for a three-clique in the relevance network is that the weakest edge (the one with the smallest mutual information) is due to indirect regulation. Therefore, this edge removed.

### GENIE3 comparison

The results of IDEM were compared to those produced by GENIE3 [22]. GENIE3 attempts to reverse engineer directed edges (though the biological interpretation of the direction is not explicitly stated) using only expression data, even though causal direction is often not identifiable from such data even under the strong assumptions of causal sufficiency and causal faithfulness. The purpose of this comparison is to demonstrate the value added by methylation data, especially when inferring directed networks. The data provided to GENIE3 was the same expression data provided to IDEM and was fully preprocessed except for the binning step. Since GENIE3 produces a weight for each edge rather than a hard decision, we considered the top *M* edges for each dataset, where *M* is the number of edges (both true and false) discovered by IDEM on the same dataset. Since GENIE3 produces a weight for both directions for every edge, only the larger of the two were considered when selecting the top *M* edges.

## Results

IDEM was applied to the TCGA datasets mentioned previously, as well as the synthetic datasets, with *B* = 2. At the sample sizes available, using *B* = 2 proved empirically more successful than larger values of *B*. (Data not shown). Since *α* = 10^-3^ provided a good balance between high precision and sufficient recall to draw meaningful conclusions, detailed analyses (all analyses except the PR curve) were performed using *α* = 10^-3^. The full network that we reverse-engineered for each dataset is available in Additional files 5 and 6.

### Synthetic data

We first attempted to validate IDEM using synthetic expression data generated using GeneNetWeaver [34-36], the software used to simulate data for the DREAM challenge. Since differential methylation constitutes a natural multifactorial perturbation experiment in our model and GeneNetWeaver does not include any facilities to simulate methylation data, we created a set of perturbations analogous to methylation data as described below. We then used GeneNetWeaver’s multifactorial perturbation feature to generate expression data with these perturbations. The perturbations were generated by a procedure that was designed to make the distribution of absolute correlations between methylation variables and corresponding expression variables similar to that observed on the real GBM and ovarian data. For each gene *g*, first generate a standard deviation *σ*_*g*_ from a uniform distribution over [0,0.05], and then generate perturbation values *m*_*g*,*j*_, *j* = 1, …, *N*, from a *Normal*(0, *σ*_*g*_) density. The matrix of perturbations (i.e., genes by samples) was supplied to IDEM as the “methylation” data.

A network of 1,000 genes was generated by randomly placing approximately 2,000 edges. Each gene *g* was assigned a random outgoing and incoming weight, which were proportional to the probabilities of each edge being outgoing from *g* and incoming to *g* respectively, or in other words proportional to the expected out and in degree of each gene. The outgoing weights were sampled from the distribution *P*(*W*) = *w*^-2^, *w* ≥ 1. The intent was for the out degree distribution to approximate a scale-free network. The incoming weights were sampled from *P*(*W*) = 2*e*^-2*w*^, *w* ≥ 0. This network is available in Additional file 7.

After the network and perturbation data were generated, the expression data was generated using GeneNetWeaver, using the stochastic differential equation model and otherwise using the default settings. The expression data used included the simulated microarray noise that GeneNetWeaver is capable of producing. The synthetic datasets are available in Additional files 8 and 9.

The above simulation was performed at sample sizes of 100, 500 and 1,000. Since the full ground truth network was available for this dataset, traditional precision and recall statistics can be used to assess the accuracy of edge placement (EP) in the reverse-engineered network. For the purpose of this benchmark, an edge is considered a true positive only if the direction is correct. The contingency table used is shown in Table 2, where *R* represents a regulator gene and *T* represents a target of a given regulator. From this table recall and precision statistics can be calculated. The recall is *TP* / (*TP* + *FN*) and the precision is *TP* / (*TP* + *FP*). The null recall, or expected recall if IDEM has no predictive ability, is (*TP* + *FP*) / *E* where *E* is the total number of edges classified. The null precision is (*TP* + *FN*) / *E*. The statistical significance of this benchmark was assessed using the one-sided version of Fisher’s Exact Test. Additionally, we measured the accuracy of edge direction (ED) prediction given that a correct undirected edge was discovered. Since complete ground truth data is available, the significance of this can be assessed by a simple one-sided binomial test. The reverse-engineered networks are available in Additional file 10.

**Table 2:** Edge placement benchmark

	**No predicted *R → T***	**Predicted *R → T***
No actual *R* → *T*	*TN*	*FP*
Actual *R* → *T*	*FN*	*TP*

Contingency table used for the edge placement (EP) benchmark. The row determines whether an edge *R* → *T* exists according to the knockdown data. The column determines whether an edge *R* → *T* is predicted. The symbols TP, TN, FP and FN stand for true positives, true negatives, false positives and false negatives respectively.

Tables 3 and 4 display the result of IDEM on the synthetic dataset. The results are compared to GENIE3 [22], a method that attempts to learn directed regulatory edges using expression data only. In terms of edge placement (Table 3), the recall is poor for both algorithms, which is frequently the case in computational methods for reverse-engineering regulatory networks; on the other hand, the precision of IDEM quite reasonable. In terms of edge direction (Table 4), IDEM is virtually perfect in identifying the direction of edges given that an edge is predicted.

**Table 3:** Synthetic data edge placement results

**Method**	**N**	**Precision**	**Null**	**Recall**	**Null**	**Fisher**
	**samples**		**prec.**		**recall**	**p-value**
IDEM	100	0.565	0.0039	0.007	4.6e-5	3.6e-28
IDEM	500	0.681	0.0039	0.032	1.8e-4	5.1e-127
IDEM	1000	0.692	0.0039	0.057	3.3e-4	6.1e-226
GENIE3	100	0.130	0.0039	0.002	4.6e-5	9.7e-5
GENIE3	500	0.165	0.0039	0.008	1.8e-4	2.7e-20
GENIE3	1000	0.119	0.0039	0.010	3.3e-4	1.5e-22

The results of the edge placement (EP) benchmark using synthetic data.

**Table 4:** Synthetic data edge direction results

**Method**	**N samples**	**Fract correct**	**Binomial p-value**
IDEM	100	1	0.001
IDEM	500	1	2.2e-19
IDEM	1000	1	7.7e-34
GENIE3	100	0.43	0.77
GENIE3	500	0.48	0.64
GENIE3	1000	0.5	0.56

The results of the edge direction (ED) benchmark using synthetic data.

### Knockdown validation

Knockdown experiments, where the expression of individual genes is perturbed in a targeted manner, can provide valuable information about regulatory networks. To the best of our knowledge, no publicly available knockdown data exists for the same tissue types for which TCGA methylation and expression data are available. We therefore evaluted IDEM’s predictions using a publicly available siRNA knockdown dataset from a human myeloid leukemia cell line [37]. This dataset was used under the assumption that gene regulatory networks are partially conserved across tissue types. It contains expression levels for control samples as well as samples with approximately 50 genes knocked down. The genes that were knocked down are referred to as “knockdown genes”. Seventeen negative control replicates were included and for most knockdown genes three replicates were included. Where multiple probe sets mapped to the same gene, the maximum expression level was used.

Since knocking down a gene is an intervention in the context of a controlled experiment, changes in a gene’s expression upon knocking down the knockdown gene are assumed to be caused by the knockdown. Therefore, we declared a target gene *T* to be regulated by a regulator *R* if *T* was differentially expressed between control samples and samples with *R* knocked down with a p-value ≤0.01 as assessed by the Wilcoxon Rank Sum Test and with a fold change greater than two between the median control and knockdown expression levels. We also required that *T* be expressed in either the control or knockdown samples with a geometric mean detection p-value ≤0.001 for the probe set used to represent each gene. This definition allows *R* to regulate *T* indirectly. Distinguishing direct from indirect regulation was not feasible given the nature of the knockdown dataset. To accommodate this ambiguity, the indirect edge removal step of the IDEM algorithm was skipped when preparing IDEM predictions for this validation.

The edge placement benchmark determines whether an *R* → *T* edge is more likely to be predicted by IDEM when *T* is differentially expressed upon knocking out *R* than when *T* is not differentially expressed. This is identical to the EP benchmark used on they synthetic data except that it is only performed for the subset of edges for which knockdown data is available. The edge direction benchmark is similar to the EP benchmark but is conditioned on IDEM predicting an edge between *R* and *T* in either direction (either *R* → *T* or *T* → *R*). The null hypothesis is that *P*_*signif*_, the probability that IDEM predicts an edge *R* → *T* given that an *R* → *T* edge exists according to the knockdown data, is equal to *P*_*non* - *signif*_, the probability that IDEM predicts *R* → *T* given that this edge does not exist according to the knockdown data. The alternative is *P*_*signif*_ >v*P*_*non* - *signif*_. This benchmark is meant to test only the accuracy of the inferred edge direction, which is the novel part of IDEM. This formulation is necessary since only approximately 50 genes were knocked down in these experiments. For most knockdown genes *R*, the majority of edges GENIE3 and to a lesser extent IDEM predict are outgoing (*R* → *T*) regardless of whether *T* is differentially expressed when *R* is knocked down. Demonstrating predictive value requires demonstrating further enrichment when *T* is differentially expressed. The contingency table used for this benchmark is shown in Table 5. *P*_*signif*_ can be written as *N*_22_ / (*N*_21_ + *N*_22_) and *P*_*non* - *signif*_ can be written as *N*_12_ / (*N*_11_ + *N*_12_). The statistical significance of this was also assessed with Fisher’s Exact Test.

**Table 5:** Knockdown data edge direction benchmark

	**Predicted *T → R***	**Predicted *R → T***
No Knockdown *R* → *T*	*N*_11_	*N*_12_
Knockdown *R* → *T*	*N*_21_	*N*_22_

Contingency table used for the edge direction (ED) benchmark. This table is conditioned on IDEM predicting that an edge exists either *R* → *T* or *T* → *R*. The row determines whether an edge *R* → *T* exists according to the knockdown data. The column determines which direction IDEM predicts for the edge.

**Figure 5:**
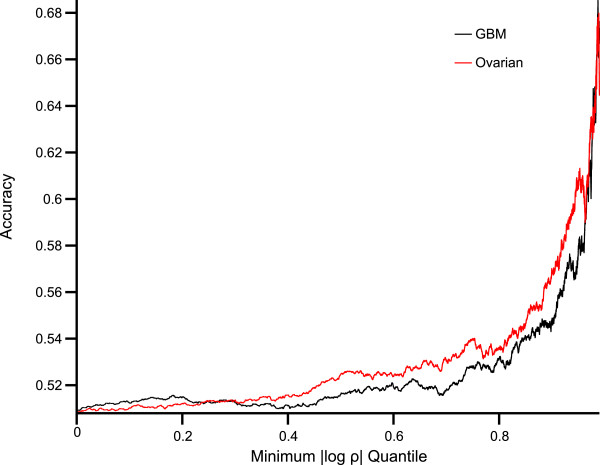
**LR Vs. Accuracy.** The accuracy of our likelihood ratio method of inferring edge direction as a function of minimum | log*ρ*| when edge placement is given. For example, among edges with | log*ρ*| in the top 1% the accuracy is approximately 67% on the GBM dataset and 64% on the ovarian dataset.

The ED benchmark measures how well IDEM predicts edge direction given that it predicts the existence of an edge. We also measured the extent to which our likelihood ratio test predicts edge direction when edge placement is given. For each edge in the knockout data as defined above, we applied IDEM’s likelihood ratio test to the methylation and expression data for the relevant gene pair to predict direction. Since larger absolute log likelihood ratio (| log*ρ*| where *ρ* is the likelihood ratio test statistic described in Methods) indicates greater confidence in the edge direction selected, we plotted the accuracy vs. minimum | log*ρ*| quantiles. Note that no non-causal pruning step is used in Figure 5, and this step tends to remove edges with small | log*ρ*|. Therefore, accuracies when little constraint is placed on | log*ρ*| (those near the left edge of Figure 5) are much lower than those observed in the ED benchmark (Table 5).

**Figure 6:**
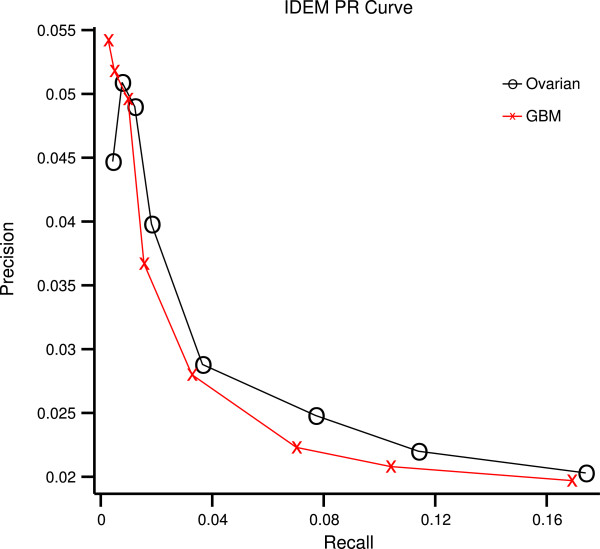
**PR curve.** A PR curve produced as described in the “Knockdown validation” section, using the real ovarian and GBM data. At the far right of the curve, the observed precision approaches the null precision (or the precision expected if edges were placed randomly. Therefore, only the portion of the curve where the observed precision is noticeably above the null precision is shown.

Tables 6 and 7 validate IDEM’s results when the knockdown data described in Methods is treated as ground truth. The results are again compared to GENIE3. The accuracy of IDEM in determining both edge placement and edge direction is significantly better than chance but still modest at available sample sizes. However, Figure 5 demonstrates that, when edge placement is given, the likelihood ratio test becomes increasingly accurate for high-confidence predictions (those with large | log*ρ*|). For example, among edges with | log*ρ*| in the top 1% the accuracy is approximately 67% on the GBM dataset and 64% on the ovarian dataset. Additionally, a precision-recall (PR) curve of IDEM’s performance on real data is shown in Figure 6.

**Table 6:** Knockdown data edge placement results

**Dataset**	**Method**	**Precision**	**Null**	**Recall**	**Null**	**Fisher**
			**prec.**		**recall**	**P-Val**
GBM	IDEM	0.0367	0.017	0.015	0.0073	1.13e-16
Ovarian	IDEM	0.0397	0.018	0.018	0.0081	7.68e-21
GBM	GENIE3	0.032	0.017	0.010	0.0057	1.76e-08
Ovarian	GENIE3	0.033	0.018	0.011	0.0058	3.87e-09

The results of the edge placement (EP) benchmark using knockdown data.

**Table 7:** Knockdown data edge direction results

**Dataset**	**Method**	***P***_***signif***_	***P***_***non-signif***_	**Fisher P-val**
GBM	IDEM	0.606	0.51	0.0019
Ovarian	IDEM	0.618	0.529	0.0025
GBM	GENIE3	0.680	0.656	0.305
Ovarian	GENIE3	0.705	0.696	0.452

The results of the edge direction (ED) benchmark using knockdown data.

### KEGG validation

We attempted to validate IDEM’s edge direction prediction on a small set of known cancer-related interactions from the KEGG [38] hsa05200 pathway. IDEM was run with *α* = 1 and DPI tolerance of 1 so that all possible edges would be predicted and only the direction of the edge remained to be reverse-engineered. The transcription factor-target interactions in this dataset overlapped with 51 IDEM interactions in the GBM dataset and 44 in the ovarian dataset. (These numbers differ due to the criteria for eliminating genes in the case of missing values.) The results are shown in Table 8.

**Table 8:** KEGG validation

**Dataset**	**N correct direction**	**N interactions**	**binomial P-Val**
Ovarian	20	44	0.774
GBM	33	51	0.024

The results of comparing IDEM’s edge direction prediction to the directions of TF-target interactions in the KEGG hsa05200 pathway.

### Computational complexity

IDEM is designed to scale computationally to large datasets. Therefore, each step is of reasonable time complexity. Let *N* be the number of samples and *M* be the number of genes. The time complexity of the binning step is *O*(*MN* log*N*). The complexity of building the relevance network is *O*(*M*^2^*N*). The time complexity of performing the likelihood ratio test is *O*(*EN*) where *E* is the number of edges remaining at this step. The time complexity of the indirect edge removal is *O*(*M*^3^) in the worst case but in practice much smaller because the graph to which it is applied is typically sparse. Every major step can also be efficiently parallelized, and the building of the relevance network, the application of the likelihood ratio test and the application of the data processing inequality are parallelized in our reference implementation. The reference D implementation takes under 10 minutes to run an 8-core Xeon X5647 machine for any dataset described.

### Theoretical results

This section proves a set of results with regard to the consistency of IDEM. Consistency means that, given infinite samples, the correct causal graph would be recovered. Since this section discusses consistency, it is assumed that, given adequate sample size, an arbitrarily large number of bins could be used for the binning process. This would asymptotically eliminate any biases due to the binning process.

### Proof of consistency of likelihood ratio test

Let

$$R_{1}=E_{P}\left(\log\frac{P(E_{1},E_{2}|M_{1},M_{2})}{P(E_{1}|M_{1})P(E_{2}|E_{1},M_{2})}\right)$$

and

$$R_{2}=E_{P}\left(\log\frac{P(E_{1},E_{2}|M_{1},M_{2})}{P(E_{2}|M_{2})P(E_{1}|E_{2},M_{1})}\right)$$

so that *E*_*P*_(*llr*) = *R*_2_ - *R*_1_.

We have

$$\begin{array}{ll} \;R_{1} & =H(E_{1}|M_{1})+H(E_{2}|E_{1},M_{2})-H(E_{1},E_{2}|M_{1},M_{2})\\ & =H(E_{1}|M_{1})+H(E_{2}|E_{1},M_{2})-H(E_{1},E_{2},M_{1},M_{2})\\ & \;+H(M_{1},M_{2})\\ & =H(E_{1}|M_{1})+H(M_{1},M_{2})-H(M_{1}|E_{1},M_{2})\\ & \;-H(E_{1},M_{2})+I(E_{2},M_{1}|E_{1},M_{2})\\ & =H(E_{1}|M_{1})+H(M_{2}|M_{1})-H(M_{1},E_{1},M_{2})\\ & \;+H(M_{1})+I(E_{2},M_{1}|E_{1},M_{2})\\ & =I(E_{1},M_{2}|M_{1})+I(E_{2},M_{1}|E_{1},M_{2}) \end{array}$$

By symmetry,

$$R_{2}=I(E_{2},M_{1}|M_{2})+I(E_{1},M_{2}|E_{2},M_{1})$$

so that

$$\begin{array}{ll} \;E_{P}(\text{llr}) & =I(E_{2},M_{1}|M_{2})+I(E_{1},M_{2}|E_{2},M_{1})\\ & \;-(I(E_{1},M_{2}|M_{1})+I(E_{2},M_{1}|E_{1},M_{2})). \end{array}$$

#### Effects of marginalization

This subsection examines the effects of marginalizing on any variables not present in {*M*_1_, *M*_2_, *E*_1_, *E*_2_} on the identifiability of the *E*_1_ - *E*_2_ edge, assuming that marginalization adds dependencies not present in Model 1 or Model 2. In this section, graphs are to be interpreted only as Bayesian networks and not as causal graphs, since the intent is to explore the effects of missing causal variables that introduce additional dependencies.

To simplify the notation, we marginalize over *V* first, which is equivalent to placing an edge between *M*_1_, *M*_2_. The direction of this edge is not important since both directions lead to the same independence relationships among {*M*_1_, *M*_2_, *E*_1_, *E*_2_}.

There are two edges that can be added to this graph while keeping it directed acyclic and without adding vertices: *M*_1_ - *E*_2_ or *M*_2_ - *E*_1_. (Since these edges appear on the diagonal in all figures in this section, we refer to them as “diagonal edges”). Again, the direction is not important as long as the directions of these edges and the *M*_1_ - *M*_2_ edge are chosen such that the graph is acyclic. The diagonal edges are not to be interpreted biologically as direct causal relations. They only model the statistical effects of marginalizing over variables excluded from Models 3 and 4. If both edges are added, the graph is fully connected. No independence relationships remain regardless of the direction of the edge between *E*_1_, *E*_2_, so this direction is unidentifiable.

**Figure 7:**
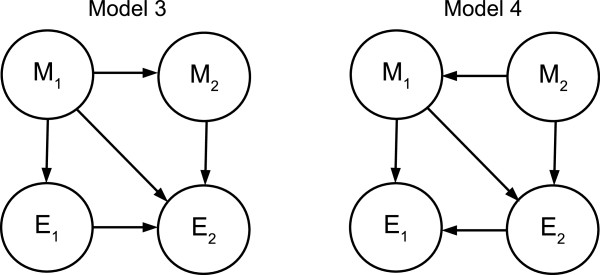
**Models 3, 4.** Bayesian network graphs for Models 3 and 4. These are not causal graphs and are intended to represent possible effects of marginalizing over variables outside the subgraph {*M*_1_, *M*_2_, *E*_1_, *E*_2_} on the independence relationships among these variables.

When one of the two diagonal edges is added, the edge direction remains identifiable but a modification of the likelihood ratio test is required. This modification requires knowing or inferring which diagonal has been added and estimation of a quadruple distribution instead of a triple. Similarly to the previous section, consider the two models illustrated in Figure 7. We consider only one possible diagonal edge. This is without loss of generality due to symmetry. The likelihood ratio here is:

(1) $$R_{34}=\frac{P(E_{1}|M_{1})P(E_{2}|E_{1},M_{1},M_{2})}{P(E_{2}|M_{1},M_{2})P(E_{1}|M_{1},E_{2})}$$

Under Model 3 *E*_1_ ⊥ *M*_2_|*M*_1_ and under Model 4 *E*_1_ ⊥ *M*_2_|*M*_1_, *E*_2_. Using notation and arguments similar to those in the previous section it can be shown that *E*_*P*_[ *R*_34_] = *I*(*M*_2_;*E*_1_|*M*_1_,*E*_2_) - *I*(*M*_2_;*E*_1_|*M*_1_).

Therefore, these models are identifiable via a likelihood ratio test unless the independence relationships implied by both models apply simultaneously.

#### Proof of consistency for acyclic causal Markov graphs

In this section we derive a useful set of sufficient conditions for IDEM to be consistent. Consistency means that, given infinite samples, the correct causal graph would be recovered. Given a sufficiently large sample size an arbitrarily large number of bins could be used for estimating mutual informations and likelihood ratios, eliminating information loss due to binning. We demonstrate that IDEM can correctly recover the any causal graph *G* where, in addition to the assumptions described in the main text, the following are true: 

1. The Causal Markov Condition applies for *G*. Most importantly, this means no common causes have been omitted [12].

2. Causal faithfulness [12,13] applies. This means that *only* the independence relationships specified by the causal graph and Causal Markov Condition exist.

3. The causal graph must be acyclic. This means no directed or undirected cycles.

4. The Data Processing Inequality must be strict. Let *A* - *B* - *C* be a Markov chain implied by a causal graph and the Causal Markov Condition. Then *I*(*A*;*C*) < min(*I*(*A*;*B*), *I*(*B*;*C*)). If *I*(*A*;*C*) = min(*I*(*A*;*B*), *I*(*B*;*C*)) then direct vs. indirect causality will not be identifiable.

This proof consists of three elements: 

1. The ARACNE [20] method is consistent in its recovery of irreducible pairwise statistical dependencies if the graph of these dependencies is a tree. (Since the graph produced by ARACNE is undirected, a tree is equivalent to an acyclic graph.) The proof can be found in the ARACNE reference. Since IDEM uses the mutual information relevance network and data processing inequality steps from ARACNE, the same logic applies to it. IDEM will also recover irreducible pairwise statistical dependences.

2. Given an acyclic graph *G* for which the Causal Markov Condition applies, irreducible statistical dependency between variables *A*, *B* as defined in [20] exists only if a causal edge exists in *G* between *A*, *B*. This is best demonstrated by enumerating cases. In an acyclic causal graph there are three possible scenarios: 

a.) There is a causal path between *A* and *B*. If this is not a direct causal path, then the *A* - *B* edge will be eliminated by the Data Processing Inequality step if the Data Processing Inequality is strict.

a.) There is no causal path between *A* and *B* but there is still statistical dependency. Then *A* and *B* must have a common cause.

Then the *A* - *B* edge will be eliminated by the Data Processing Inequality step.

a.) There is no causal path between *A* and *B* and no common cause. *A* will then be independent of *B*.

The causal faithfulness assumption requires that an irreducible statistical dependency exists if a causal edge exists. These first two elements then prove that the correct undirected causal graph can be recovered.

**Figure 8:**
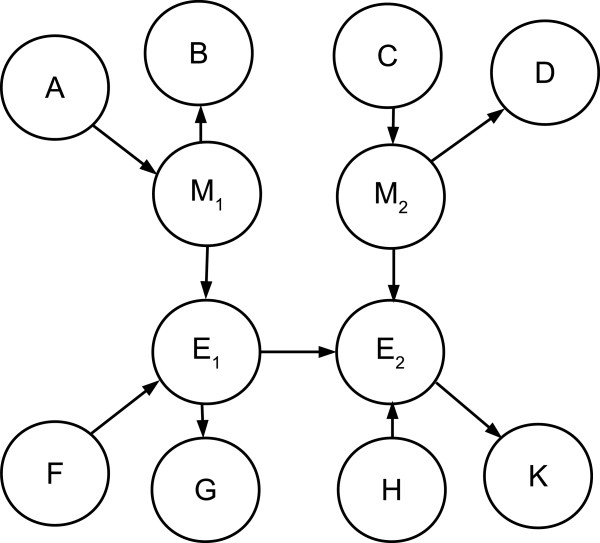
**General acyclic model.** The most general acyclic model where *E*_1_ → *E*_2_, *M*_1_ regulates *E*_1_ and *M*_2_ regulates *E*_2_. Under this model both of the independence assumptions of Model 1 as depicted in Figure 3 hold.

3. If there are no cycles the likelihood ratio test as described previously is consistent with respect to edge direction between *E*_1_, *E*_2_. The degenerate case cannot occur under causal faithfulness. Assume without loss of generality that for some set of variables {*E*_1_, *E*_2_} the edge direction is *E*_1_ → *E*_2_. Adding the respective methylation variables and all possible connections that {*M*_1_, *M*_2_, *E*_1_, *E*_2_} might have to the larger causal graph yields the graph shown in Figure 8. This is the most general acyclic model since the variables in {*A*, *B*, *C*, *D*, *F*, *G*, *H*, *K*} may be multidimensional and cannot be connected to each other without forming a cycle. Under this model the two independence relationships that apply to Model 1 as depicted in Figure 3 apply to {*M*_1_, *M*_2_, *E*_1_, *E*_2_}, so the likelihood ratio test is consistent.

This set of conditions is more general than it appears at first glance. While the complete causal graph of all genes is almost certainly not acyclic, some subgraphs may be. If no two nodes in this subgraph have a common cause outside the subgraph then the Causal Markov Condition applies and if all other conditions are met IDEM will produce consistent results.

## Discussion

To the best of our knowledge, this work is the first attempt to mitigate the problem of identifying edge direction in gene regulatory networks using only high-throughput, non-time series, observational data. The performance of the algorithm on synthetic data using the GeneNetWeaver simulator is excellent. However, the discrepancy between the accuracy of IDEM as assessed by synthetic data vs. real expression, methylation and knockdown data is substantial. The knockdown benchmark results should be taken as a lower bound on the performance of IDEM. The knockdown data comes from a different cell type than those available in TCGA. Typically only three replicates are available for each knockdown experiment, decreasing the power to infer weak regulation. To compensate we considered differential expression statistically significant if the p-value was ≤0.01 without adjusting for multiple testing. Therefore, a significant number of edges in our “ground truth” data are likely false positives. Furthermore, it is possible that a substantial number of edges inferred from the knockdown data are the result of batch effects. Finally, since the knockdown data does not allow direct vs. indirect regulation to be distinguished, the indirect edge pruning step of IDEM is not used for this benchmark. Weak, indirect edges may be much harder to reverse-engineer in practice than strong, direct edges. Nonetheless our highest confidence edge direction predictions achieve an accuracy of 64–67% using only non-time series observational data. Likewise, IDEM’s performance in correctly predicting edge direction between members of the Pathways in Cancer KEGG gene set also represents a lower performance bound. This pathway represents gene relationships described across a multitude of experiments in many different cancer systems. As a result, it is likely that many of these edges are weak or non-existant in our test datasets, hampering IDEM’s ability to correctly infer directionality.

The consideration of only pairwise and (for pruning indirect edges) three-way interactions can clearly lead to biases, especially in the case of loopy networks. A significant difficulty, detailed in Theoretical results, is that marginalization over variables outside the {*M*_1_, *M*_2_, *E*_1_, *E*_2_} subnetwork might add statistical dependencies not suggested by Model 1 or Model 2 and make our likelihood ratio test inconsistent. However this is not an issue for acyclic subgraphs for which the Causal Markov Condition holds. Furthermore, in some cases a more complex likelihood ratio test can work around the marginalization issue even while involving only variables in {*M*_1_, *M*_2_, *E*_1_, *E*_2_}. Examining higher- order joint probabilities would increase the amount of data required to maintain a constant level of estimation error by an exponential factor in the dimensionality of the interactions examined. Empirically, despite the biases introduced by low-order analysis, accuracy of more traditional pairwise methods on real and simulated data is comparable to the accuracy of methods that use higher-order analyses [39].

It is important to note that, in our network, an edge need not represent direct transcription factor binding. The reverse-engineered network is a purely phenomenological prediction of what genes would be affected if the mRNA expression of a given gene were perturbed. For example, consider a hypothetical gene *K*. When *K* is expressed, it produces a kinase protein that interacts at the protein-protein level with a constitutively expressed transcription factor protein *F*. When *F* is phosphorylated, it activates or inhibits the transcription of a target gene *T*. Since *F* is constitutively expressed, its expression will not have high mutual information with any other gene’s expression. Biologically, the gene most relevant in explaining variation in the expression of *T* is *K*. In our method, *K* will have large mutual information with *T* and the edge *K* – *T* will likely be inferred. This edge does not represent direct transcription factor-target binding but is nonetheless biologically meaningful in that perturbing *K* would affect the expression of *T* with no other mRNA concentrations being affected as a necessary condition. Similarly, due to our lack of either expression or methylation data for about half of all known genes, an edge placed between two available genes might physically involve a third, unmodeled gene as an intermediary.

The primary practical use for IDEM will likely be generating hypotheses about the nature of human disease states, or treatment targets for such diseases. A significant benefit to the methodology is that the amount of publicly available joint methylation and mRNA expression data is rapidly increasing. As such data increases in availability, various datasets can be combined to produce a network with increasing sensitivity. Two limitations of IDEM are i)the use of methylation-induced epigenetic silencing to provide context for reverse-engineering directed edges precludes the use of this method in lower organisms in which methylation-induced silencing is not widely used; and ii) a graph of a regulatory network, built from publicly available data, does not provide clear conclusions on its own, but provides a useful starting point from which further studies can be undertaken to confirm and quantify the results.

Moving forward, several expression context variables in addition to methylation can be used to make edge direction identifiable. Methylation was used because it was the most practical at this time. However, variables such as copy number and the concentrations of highly targeted microRNAs can also be used. In principle, gene sets of higher order than pairs could also be considered given sufficient data and computational power. Considering higher order interactions would allow situations such as XOR logic to be discovered and remove some inconsistencies from likelihood ratio test for direction in loopy scenarios.

## Conclusions

We demonstrate the feasibility of using DNE methylation data to make directed gene regulatory edges statistically identifiable from non-time series observational data. This is shown both theoretically and empirically, on both synthetic and real data.

## Competing interests

The authors declare that they have no competing interests.

## Authors’ contributions

DS conceived the idea, performed the experiments and wrote the manuscript. LY contributed to the theoretical analysis and wrote portions of the manuscript. MA supervised the analysis of methylation data and conceived the KEGG experiment. DG conceived the statistical design, supervised the work and helped write the manuscript. All authors read and approved the final manuscript.

## Supplementary Material

Additional file 1 Preprocessed TCGA ovarian mRNA expression data. Click here for file

Additional file 2 Preprocessed TCGA glioblastoma mRNA expression data. Click here for file

Additional file 3 Preprocessed TCGA ovarian methylation data. Click here for file

Additional file 4 Preprocessed TCGA glioblastoma methylation data. Click here for file

Additional file 5 **The reverse-engineered network (*B *** **= 2, α = 0.001) for the TCGA ovarian data.** Click here for file

Additional file 6 **The reverse-engineered network (*B *** **= 2, α = 0.001) for the TCGA glioblastoma data.** Click here for file

Additional file 7 The actual network for the synthetic data. Click here for file

Additional file 8 Preprocessed synthetic expression data at each sample size. Click here for file

Additional file 9 Preprocessed synthetic “methylation” data at each sample size. Click here for file

Additional file 10 **The reverse-engineered network (*B *** **= 2, α = 0.001) for the synthetic data at each sample size.** Click here for file
